# Normal Action of Therapeutic Agents

**Published:** 1873-07

**Authors:** 


### NORMAL ACTION OF THERAPEUTIC AGENTS.

As we desire to make a consideration of therapeutic agents with respect to their normal action, as well as therapeutical results, one of the features of the JOURNAL, we solicit contributions on this subject, in reference especially to discoveries in the direct impression made by remedies upon particular parts of the body, and the kind of action exerted. The advancement of rational medicine will certainly be promoted by the publication of such facts. In order to a knowledge of the practical use of curatives, their normal action must be understood. We would not, however, exclude records of the therapeutical results of remedies empirically given; for often in this way their direct effects are ascertained. Cinchona, for example, was for a long time prescribed without a correct understanding of its *modus operandi* in the cure of malarious fever. It was finally suggested that its influence is exerted upon the spinal nervous center, and that this normal action upon the cord relieves it from the depressing influence of miasmatic poison. And thus in ascertaining the direct action of bark, some important light has been thrown upon the pathology of remittent and intermittent fevers.

The value of reports would be greatly enhanced should contributors think proper to give their opinion of the *modus operandi* of remedies whose use is followed by relief. Moreover, normal action being that which is exerted upon a particular part, whether in health or disease, statistics of experiments made upon lower animals, and on the human subject, which may often with propriety be had, tend greatly toward perfecting our knowledge of the action of remedies.

